# Germline epigenetics, and reprogramming in zebrafish early embryos

**DOI:** 10.1186/1756-8935-6-S1-O23

**Published:** 2013-03-18

**Authors:** Magdalena E Potok, David A Nix, Timothy J Parnell, Bradley R Cairns

**Affiliations:** 1 Howard Hughes Medical Institute, Department of Oncological Sciences, Huntsman Cancer Institute, University of Utah School of Medicine, Salt Lake City, UT 84112, USA

## 

Early vertebrate embryos must achieve totipotency and prepare for zygotic genome activation (ZGA). To better understand, we determined DNAme profiles of zebrafish gametes, multiple embryo stages flanking ZGA, and somatic muscle - and compared them to gene activity and histone modifications. First, sperm chromatin patterns are virtually identical to those at ZGA. Unexpectedly, in the oocyte many genes important for germline functions (ie. piwil1) or early development (ie. hox genes) are DNA methylated - yet demethylated during zygotic/cleavage stages to precisely the state observed in sperm. Remarkably, this cohort constitutes the genes/loci that acquire DNAme during development (ie. ZGA to muscle). Furthermore, DNA methyltransferase inhibition experiments suggests that DNAme silences particular gene/chromatin cohorts at ZGA, preventing their precocious expression. Thus, zebrafish appear to achieve a 'totipotent' chromatin state at ZGA through paternal genome competency, maternal genome DNA demethylation/reprogramming, and the imposition of DNA methylation on genes needed later in development.

